# Influence of the Mediterranean Diet on 25-Hydroxyvitamin D Levels in Adults

**DOI:** 10.3390/nu12051439

**Published:** 2020-05-16

**Authors:** Luigi Barrea, Giovanna Muscogiuri, Daniela Laudisio, Gabriella Pugliese, Giulia de Alteriis, Annamaria Colao, Silvia Savastano

**Affiliations:** 1 Dipartimento di Medicina Clinica e Chirurgia, Unit of Endocrinology, Federico II University Medical School of Naples, Via Sergio Pansini 5, 80131 Naples, Italy; giovanna.muscogiuri@gmail.com (G.M.); dani.laudisio@libero.it (D.L.); robiniapugliese@gmail.com (G.P.); dealteriisgiulia@gmail.com (G.d.A.); colao@unina.it (A.C.); sisavast@unina.it (S.S.); 2 Centro Italiano per la cura e il Benessere del Paziente con Obesità (C.I.B.O), Department of Clinical Medicine and Surgery, Endocrinology Unit, University Medical School of Naples, Via Sergio Pansini 5, 80131 Naples, Italy; 3 Cattedra Unesco “Educazione alla Salute e allo Sviluppo Sostenibile”, Federico II University Medical School of Naples, Via Sergio Pansini 5, 80131 Naples, Italy

**Keywords:** Mediterranean diet, Vitamin D, obesity, body mass index (BMI), gender-differences, nutritionist

## Abstract

The Mediterranean diet (MD) is a dietary pattern effective in terms of prevention of obesity-related diseases, and represents the gold standard in preventive medicine, due to the synergistic action of many nutrients with antioxidant and anti-inflammatory properties. In addition, excess body weight significantly increases the risk of hypovitaminosis D, a well-recognized common feature of individuals with obesity. It is well-known that there is a clear gender difference in the adherence to the MD. The aim of this study was to investigate the association between adherence to the MD and 25-hydroxyvitamin D (25OHD) levels in adults, according to gender. Study population consisted of 617 participants; 296 were males and 321 were females, matched by age and body mass index (BMI). A validated 14-item questionnaire PREDIMED (Prevención con dieta Mediterránea) was used for the assessment of adherence to the MD. The 25OHD levels were determined by a direct competitive chemiluminescence immunoassay. Females have a higher PREDIMED score than males (7.4 ± 2.8 vs. 6.7 ± 3.1 score, *p* = 0.001), and according to PREDIMED categories, a greater percentage of males had low adherence to the MD compared to their female counterparts (40.2% vs. 37.1%; χ^2^ = 8.94, *p* = 0.003). The 25OHD levels were higher in males than in females (18.3 ± 7.3 vs. 16.8 ± 7.8 ng/mL, *p* = 0.01), and a higher percentage of males had sufficient 25OHD levels (>30 ng/mL) than their female counterparts (10.5% vs. 3.4%, χ^2^ = 10.96, *p* < 0.001). Stratifying the sample population according to 25OHD categories, BMI decreased and PREDIMED score increased significantly along with the increased 25OHD levels, in both males and females, respectively (*p* < 0.001). Looking at the bivariate correlations, PREDIMED score was positively correlated with 25OHD levels after adjusting for age and BMI, in both males (r = 0.21, *p* < 0.001) and females (r = 0.30, *p* < 0.001). At the bivariate proportional odds ratio (OR) model, 25OHD levels presented the highest OR values in the category low adherence vs. high adherence to the MD, in both genders (OR 1.21 and OR 1.31, in males and females, respectively). Receiver operator characteristic (ROC) analysis was performed to determine the cut-off values of PREDIMED scores predictive of 25OHD levels: PREDIMED score >5 in males (*p* < 0.001) and >7 in females (*p* < 0.001) could serve as thresholds for 25OHD levels above the median. The results of our study highlighted a novel positive association between adherence to the MD and 25OHD levels in both genders. Although 25OHD levels were higher in males than females, 69.7% were deficient. To the best of our knowledge, this is the first study to show that high adherence to the MD is associated with low BMI and high 25OHD levels in both genders, probably through the anti-inflammatory and anti-oxidant effects that are synergistically exerted by either MD or vitamin D on body weight.

## 1. Introduction

Hypovitaminosis D and obesity have concomitantly reached epidemic levels worldwide [1,2]. Clinical evidence reported that hypovitaminosis D represents an increasingly widespread phenomenon in patients with obesity [3,4] and it is considered a clinical syndrome due to low serum 25-hydroxyvitamin D (25OHD) levels. [1,2]. It is well-established that 25OHD levels are about 20% lower in subjects with obesity than people of normal weight [5], and the prevalence of 25OHD deficiency is greater in populations with obesity [6].

Several mechanisms have been hypothesized to explain the hypovitaminosis D in obesity, such as lower dietary vitamin D intake, lesser skin exposure to sunlight (due to reduced outdoor physical activity), reduced intestinal absorption, and accumulation of vitamin D in adipose tissue [7,8]. Different studies also indicate a strong negative association between hypovitaminosis D and several obesity-related diseases, including type 2 diabetes, cardiovascular disease, sleep disturbance related to obstructive sleep apnoea syndrome, and neurological diseases [9,10,11,12,13], suggesting that the 25OHD deficiency may play a crucial role in the pathogenesis of these diseases. In the context of weight excess, on the one hand, recent studies showed a causal role of obesity in determining 25OHD deficiency [14]. On the other hand, evidence has reported that obesity is associated with hypovitaminosis D [14,15] and suggested that low vitamin D status could affect health risks in obesity [9].

Interestingly, diet play a key role in obesity, and different dietary strategies, including MD, can reduce weight [16,17]. In addition, excess body weight significantly increases the risk of hypovitaminosis D, a well-recognized common feature of individuals with obesity [1,7,8]. The Mediterranean Diet (MD) is characterized by plant-based foods, including legumes, green-leafy vegetables, fruits, and nuts; high consumption of fish and low of red meat; moderate consumption of dairy products; and low consumption of red wine with meals (if already consuming alcohol). In addition, it includes the use of extra virgin olive oil as the main source of dietary fat. Indeed, it is the synergistic consumption of these Mediterranean foods which exemplifies the antioxidant and anti-inflammatory nature of this diet pattern [18].

It has been well reported that the MD prevents weight gain and obesity [16,19,20], and this has been attributed to its antioxidant and anti-inflammatory properties [16,21,22]. In addition, Castro-Quezada I et al. has recently reported that a higher adherence to the MD was associated with a higher prevalence of subjects showing adequate intake of nutrients, including vitamin D [23]. Other previous studies demonstrated that subjects with higher adherence to the MD also had higher dietary vitamin D intake [24,25,26].

In particular, Marventano S et al. reported the difference of nutrient intake according to quartiles of adherence to the MD and showed that dietary vitamin D intake was significantly different between the lowest and the highest quartile (4.4 vs. 6.4 µg, *p* < 0.001; respectively) [25]. Of interest, in Italy, the recommendation in both males and females is 15 µg/day (18–54 age) [27], an amount that is usually not achieved either from Western diets [28] or from a high adherence to the MD [25].

Only the HELENA study of 2330 European adolescents reported that adherence to the MD evaluated by the adapted MD quality index (KIDMED) was associated not only with better nutrient intakes, but also with higher levels of some nutritional biomarkers, including 25OHD [29]. Otherwise, to the best of our knowledge, no studies evaluated the association between the adherence to the MD and 25OHD levels in adults.

Of interest, it is well known that non-modifiable factors, including gender, influence food preferences and consumption amounts [30]. In particular, scientific evidence reported that females have a dietary profile characterized by a higher intake of fruits and vegetables than males, who consume more meat, eggs, and dairy products [31,32]. Beyond individual food choices, recent studies have reported that females showed an overall higher adherence to the MD compared with their male counterparts [33,34,35]. In addition, results of a large epidemiological study, The Nurses’ Health Study by Heidemann C et al., clearly indicated that greater adherence to a “prudent pattern,” characterized by a high intake of vegetables, fruit, legumes, fish, poultry, and whole grains, was related to a lower risk of cardiovascular and total mortality in healthy women [36]. Very recently, we have reported that 25OHD levels were lower in females than in males across all body mass index (BMI) categories [37]. As far as we know, evidence on a gender difference in the association between a healthy eating pattern, such as MD, and 25OHD levels, is still lacking. Considering the influence of the adherence to the MD on the weight excess and 25OHD levels, and the potential effects of gender on these variables, we designed the present cross sectional, single centre study to investigate the possible gender-specific association between adherence to the MD and 25OHD levels in a sample of adult population across BMI categories.

## 2. Material and Methods

### 2.1. Design and Setting

This cross-sectional, observational, single center study was performed at the Unit of Endocrinology, Department of Clinical Medicine and Surgery, Federico II University Naples (Italy), from October 2016 to January 2020. The study ws carried out taking into account the Code of Ethics of the World Medical Association (Declaration of Helsinki) for experiments involving humans. The protocol was formal approved by the Ethical Committee of the Federico II University of Naples, Medical School (number 173/16). Every enrolled subject provided informed consent after a thorough explanation of the protocol.

### 2.2. Population Study

Recruitment strategies included 617 adult Caucasians subjects (18–57 years) of both genders consecutively enrolled among patients in Endocrinology Unit, hospital volunteers, and employees. All participants were enrolled only in the autumn or winter seasons (October to March) and all were from the same geographical area around the Naples metropolitan area, Italy (latitude 40°49′ N; elevation 17 m). Therefore, all subjects had similar sun exposure potential. In addition, none of the participants reported sun exposure at the time of enrollment and were engaged in leisure time physical activity. All female subjects were non-pregnant and non-lactating, and were evaluated in the follicular phase of the menstrual cycle. A full medical history, including drug use, was collected. In order to increase the homogeneity of the subject samples, we included only adults of both genders passing the following criteria of exclusion:

- Specific nutritional regimens or hypocaloric diet in the last three months, including vegan or vegetarian diets.
- Vitamin/mineral or antioxidant supplementation.
- Weight-loss medications.
- Current therapy with calcium, vitamin D supplementation, or osteoporosis therapies; medications that may affect vitamin D absorption or metabolism, including anti-inflammatory drugs, sex hormone therapy, statins, and other hypolipidemic agents.
- Underweight patients with BMI <18.5 kg/m^2^.
- Altered thyroid hormone function tests or thyroid hormone treatment.
- Presence of type 2 diabetes mellitus (defined by criteria of the American Diabetes Association as follows: basal plasma glucose level ≥126 mg/dL on two occasions, or glycated haemoglobin ≥6.5% (≥48 mmol/moL) on two occasions, or both at the same time. Participants on antidiabetic medication were considered to have type 2 diabetes mellitus.
- Chronic diseases that could interfere fluid homeostasis, such as liver or renal chronic diseases, cancer, and acute or chronic inflammatory diseases.
- Altered levels of serum creatinine, serum calcium, or albumin.

### 2.3. Power Size Justification

The power of the sample was calculated by the differences of means ± standard deviations (SDs) of the Prevención con dieta Mediterránea (PREDIMED) score and 25OHD levels in males and females (18.3 ± 7.3 vs. 16.8 ± 7.8 ng/mL and 6.7 ± 3.1 vs. 7.4 ± 2.8 scores; respectively). When considering the number of cases required in each group of 249 was set at 296 for males and 321 for females, a type I (alpha) error of 0.05 (95%), and a type II (beta) of 0.05, the calculated power size was 95%. The calculation of sample size and power were performed while using a sample size calculator Clinical Calc [38], as previously reported [39,40,41].

### 2.4. Anthropometric Measurements

Measurements were performed in the morning, between 8 am and 10 am, after an overnight fast, by a single nutritionist. The anthropometric measurements were performed following standard criteria by the same nutritionist according to the International Society for the Advancement of Kinanthropometry (ISAK 2006). The subjects were recommended to dress light clothes and to remove shoes during the assessment, as previously reported [42,43,44,45,46,47].

BMI (weight (kg) divided by height squared (m^2^), kg/m^2^) was calculated after measuring weight and height. A wall-mounted stadiometer (Seca 711; Seca, Hamburg, Germany) was used to measure height while a calibrated balance beam scale (Seca 711; Seca, Hamburg, Germany) was used to assess weight. The degree of obesity was established according to World Health Organization WHO’s criteria: BMI: 18.5–24.9 kg/m^2^, normal-weight; BMI: 25.0–29.9 kg/m^2^, overweight; BMI: 30.0–34.9 kg/m^2^, grade I obesity; BMI: 35.0–39.9 kg/m^2^, grade II obesity; BMI ≥40.0 kg/m^2^, grade III obesity [48].

### 2.5. Assay Methods

Samples were collected in the morning between 8 am and 10 am, after an overnight fast of at least 8 h, and stored at −80 °C until being processed. The 25OHD levels were quantified by a direct competitive chemiluminescence immunoassay (CLIA) (Liaison^®^, DiaSorin, Saluggia, Italy), with a specificity of 100% for 25OHD. The analytical measurement range of detection is 4–150 ng/mL, whereas the intra-assay coefficients of variation (CVs) were 5.4%, 2.8%, and 4.7%, and the inter-assay CVs were 10.1%, 4.8%, and 7.9% for low, medium, and high points of the standard curve, respectively; as previously reported [49,50]. A 25OHD deficiency was defined as 25OHD levels <20 ng/mL (50 nmol/L); insufficiency was between 21 and 29 ng/mL (from 52.5 to 72.5 nmol/L); and normal levels were ≥30 ng/mL (75 nmol/L) [51].

### 2.6. Adherence to the MD

The adherence to the MD was evaluated using the previously validated 14-item questionnaire for the assessment of PREDIMED [52]. A qualified nutritionist administered the questionnaire during a face-to-face interview with all the enrolled subjects. Briefly, each item was assigned a score 1 or 0; PREDIMED score was calculated as follows: 0–5, lowest adherence to the MD; score 6–9, average adherence to the MD; score ≥ 10, highest adherence to the MD [52], as extensively reported [34,53,54,55,56].

### 2.7. Statistical Analysis

The data distribution was evaluated by Kolmogorov–Smirnov test; only age had a non-normal distribution, and it was normalized by logarithm. Skewed age variable was back-transformed for presentation in tables. Results were expressed as means ± SDs and categorical variables (BMI categories: normal-weight, overweight, grade I obesity, grade II obesity, and grade III obesity; PREDIMED categories: low adherence to the MD, average adherence to the MD, and high adherence to the MD; and 25OHD categories: deficit, insufficiency, and sufficiency) were expressed as percentages.

Differences between males and females were analyzed by Student’s independent *t*-test; the chi square (χ^2^) test was used to determine the significance of differences in frequency distribution of BMI categories, PREDIMED categories, and 25OHD categories. Differences in PREDIMED score and 25OHD levels in the BMI categories, PREDIMED categories, and 25OHD categories were analyzed by ANOVA test, with the Bonferroni test as a *post-hoc* test. The correlations among PREDIMED score, 25OHD levels, age, and BMI were assessed with the Pearson *r* correlation coefficients. Two partial correlation analyses were used to determine the relationships between PREDIMED score and 25OHD levels controlling for age and BMI, in males and females, respectively. Bivariate proportional odds ratio (OR) OR models, 95% interval confidence (IC), R^2^, Wald test, standard error, *p*-heterogeneity, and *p*-trend [57], were checked to assess the association between 25OHD levels with PREDIMED categories, as extensively reported [41,58,59]. In addition, a multiple linear regression analysis model (stepwise method), expressed as R^2^, beta (β), and *t*, with 25OHD levels as dependent variables was used to estimate the predictive value of PREDIMED score, BMI, sex, and age. Receiver operator characteristic (ROC) curve analysis was carried out in order to identify sensitivity and specificity, area under the curve (AUC), IC, and cut-off values of PREDIMED score in detecting the highest 25OHD levels. Test AUC for ROC analysis was also calculated, and we entered 0.76 for AUC ROC and 0.5 for null hypothesis values. An Alfa α level of 0.05 (type 1 error) and a β level of 0.2 (type II error) were used as the cut-off values for statistical significance. Variables with a variance inflation factor (VIF) >10 were excluded in order to avoid multicollinearity. Values ≤5% were considered statistically significant. Data were collected and analyzed using the MedCalc^®^ package (Version 12.3.0 1993–2012—Mariakerke, Belgium).

## 3. Results

Study population consisted of 617 participants; 296 were males and 321 females, matched by age and BMI. Age, anthropometric characteristics, adherence to the MD, and 25OHD levels in the whole study population and stratified by gender, are given in Table 1. Females have a higher PREDIMED score than males (*p* = 0.001), and according to PREDIMED categories, a greater percentage of males were low adherent to the MD compared to their female counterparts (*p* = 0.003). The 25OHD levels were higher in males than in females (*p* = 0.01), and a higher percentage of males had sufficient 25OHD levels (>30 ng/mL) than their female counterparts (*p* < 0.001).

The PREDIMED score and 25OHD levels in the population study across BMI categories are shown in Table 2. As reported, when stratifying the sample population according to the BMI categories, there were significant differences in both PREDIMED score and 25OHD levels; in particular, both PREDIMED score and 25OHD levels decreased significantly along with the increase of BMI (*p* < 0.001).

When analysing the response frequency of dietary components included in the PREDIMED questionnaire in detail, we found that the males consumed more extra-virgin olive oil (*p* = 0.025) and red/processed meats (*p* < 0.001), as compared with the females (Table 3).

**Figure 1:**
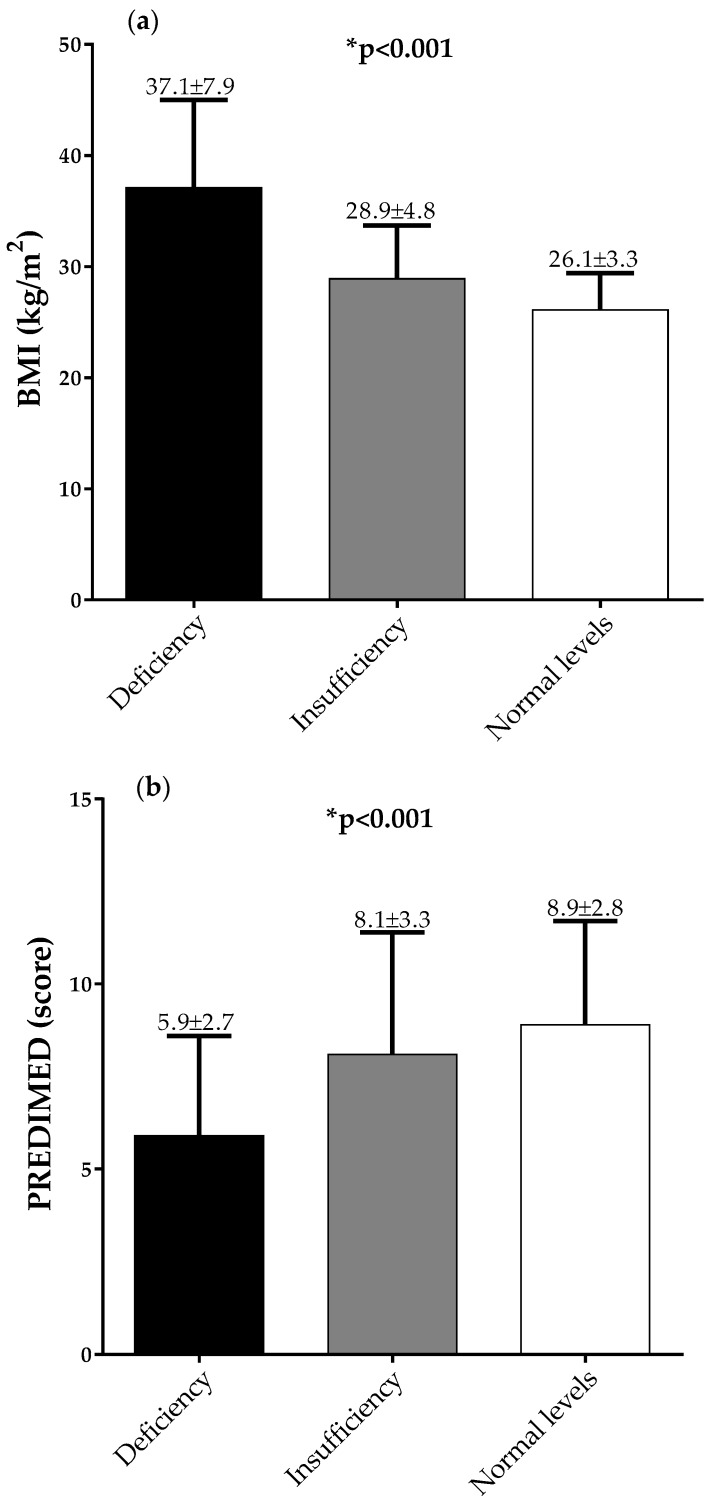

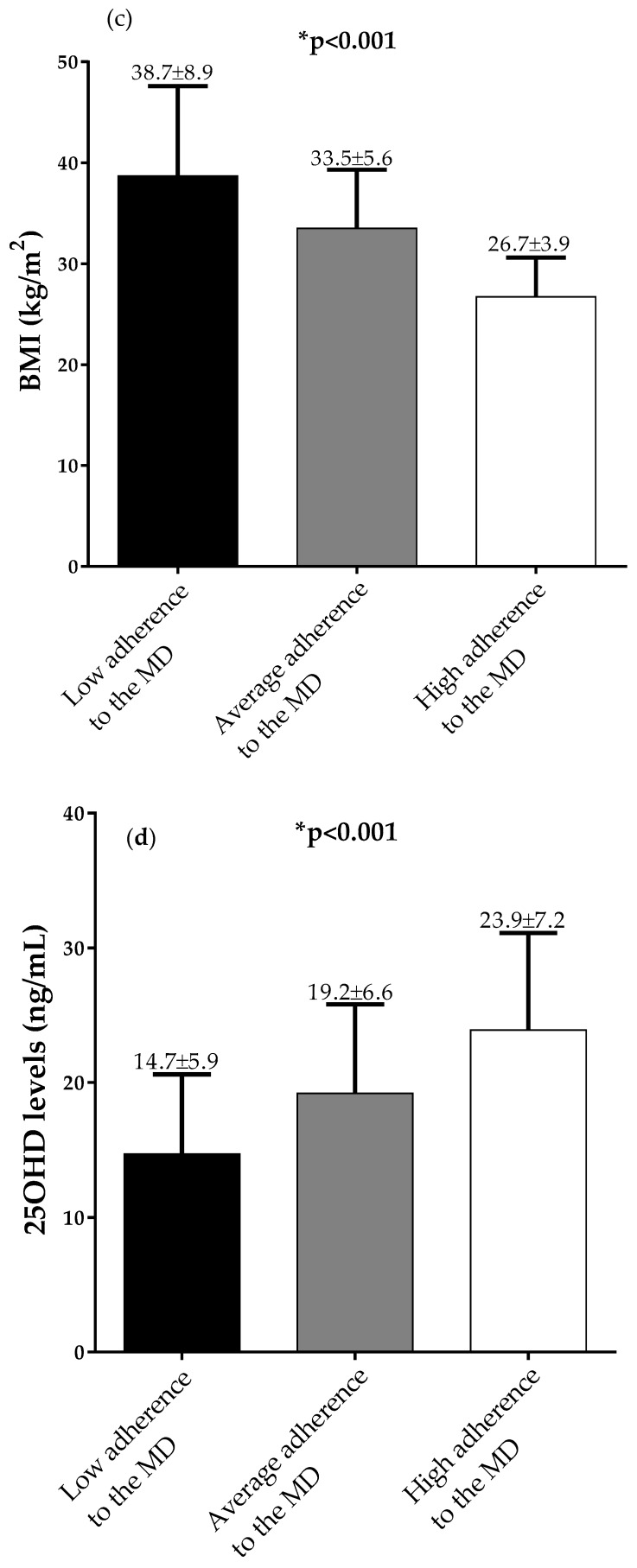
BMIs (**a**) and PREDIMED scores (**b**) across 25OHD categories, and BMIs (**c**) and 25OHD levels (**d**) across PREDIMED categories, in males. A * *p* value denotes a significant difference (*p* < 0.05).

**Figure 2:**
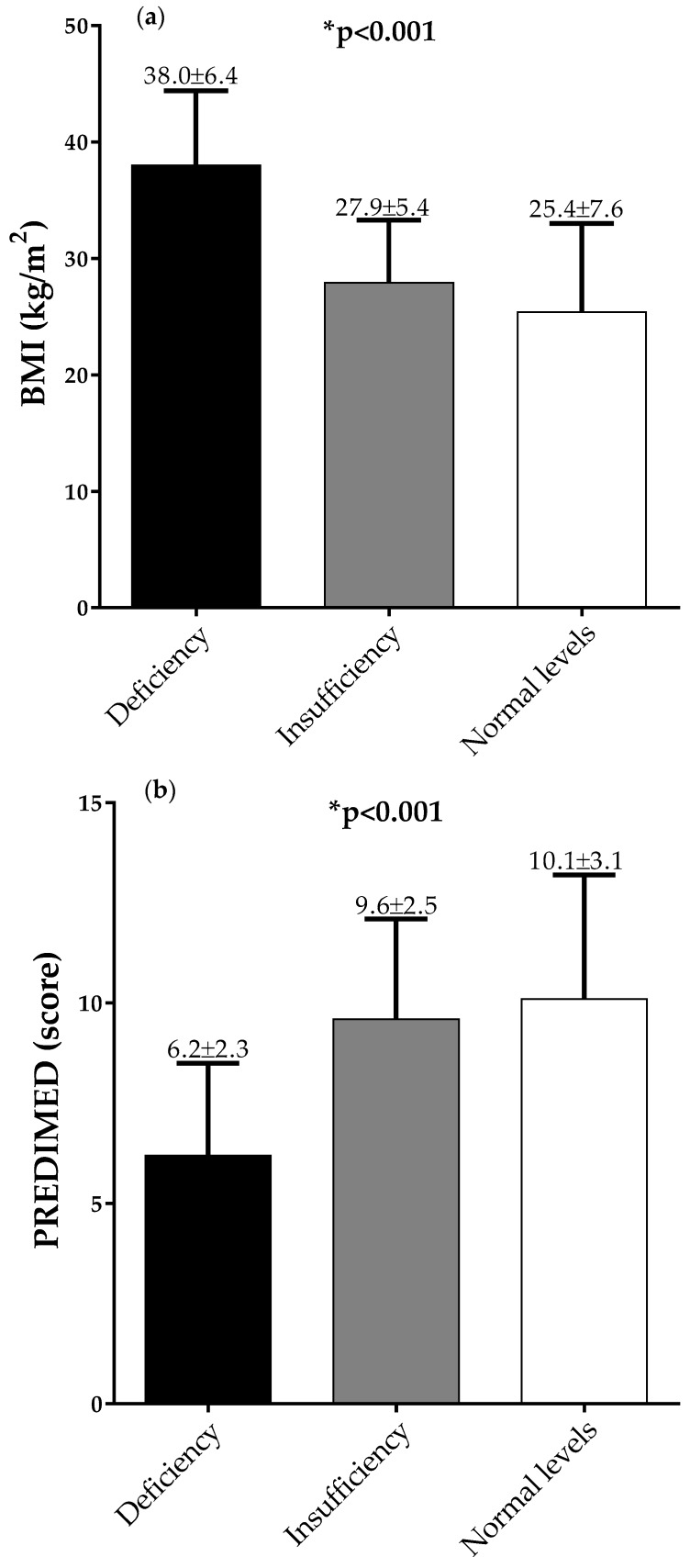

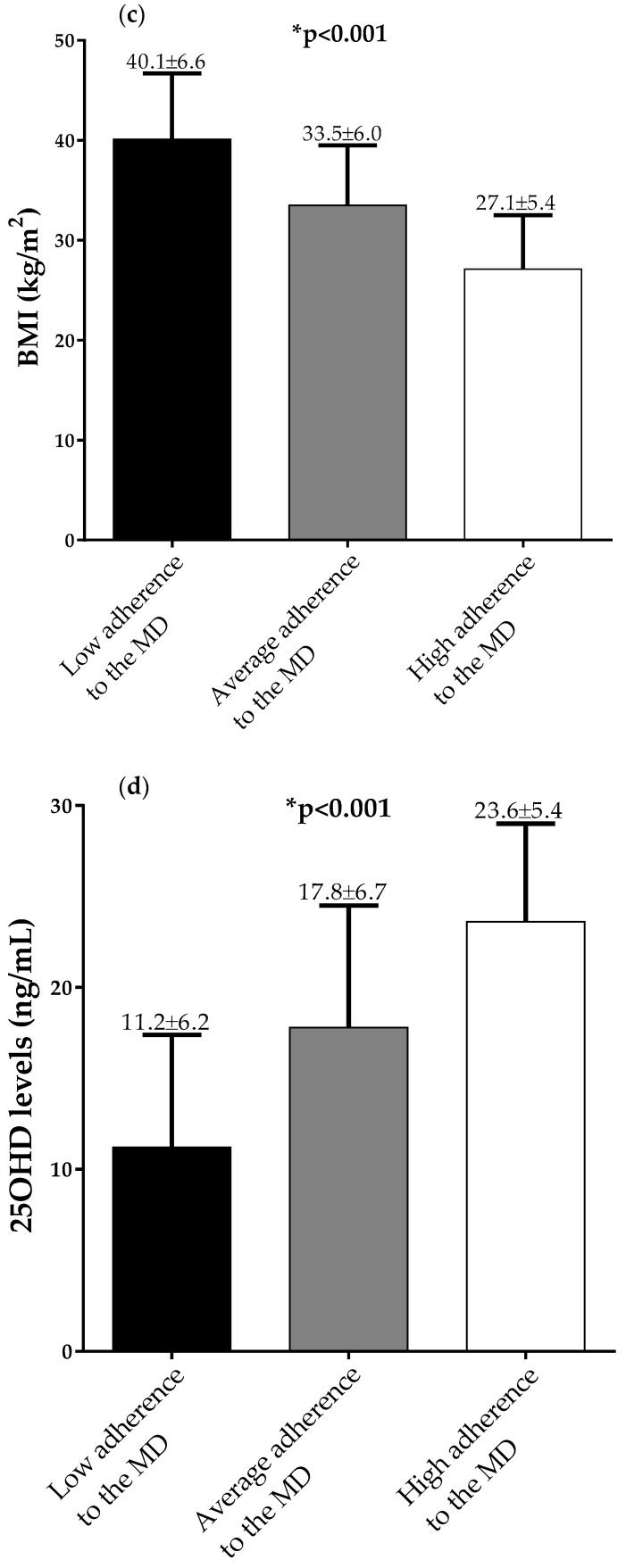
BMIs (**a**) and PREDIMED scores (**b**) across 25OHD categories, and BMIs (**c**) and 25OHD levels (**d**) across PREDIMED categories, in females. A * *p* value denotes a significant difference (*p* < 0.05).

Figure 1 and Figure 2 report BMIs (Figure 1a and Figure 2a) and PREDIMED scores (Figure 1b and Figure 2b), in males and females, respectively, across 25OHD categories. The same figures report BMI (Figure 1c and Figure 2c) and 25OHD levels (Figure 1d and Figure 2d), in males and females, respectively, across PREDIMED categories. In detail, stratifying the sample population according to 25OHD categories, BMI decreased and PREDIMED score increased significantly along with the increase in 25OHD levels (*p* < 0.001), in both males and females, respectively (Figure 1a and Figure 2a). Likewise, dividing the sample population according to PREDIMED categories, BMI decreased and 25OHD levels increased significantly, along with the increase in the adherence to the MD (*p* < 0.001), in both males and females, respectively (Figure 1b and Figure 2b).

Table 4 reported the descriptive data of PREDIMED score and 25OHD categories according to PREDIMED categories and stratified by gender. PREDIMED scores were higher in females than in males in the low and high PREDIMED categories (*p* < 0.001 and *p* = 0.03, respectively).

### Correlation Analysis

Both PREDIMED score and 25OHD levels were negatively correlated with BMI (r = −0.62 and r = −0.67, *p* < 0.001; respectively), but 25OHD levels only showed a negative correlation with age (r = −0.09, *p* = 0.02). The PREDIMED score was positively correlated with 25OHD levels (*p* < 0.001). This association was maintained after adjusting for age and BMI, in both males (r = 0.21, *p* < 0.001) and females (r = 0.30, *p* < 0.001).

The results of the bivariate proportional OR model used to assess the association of 25OHD levels with PREDIMED categories are reported in Table 5. The 25OHD levels presented the highest OR values in the category low adherence vs. high adherence to the MD, in both genders (OR 1.21 and OR 1.31, in males and females, respectively).

To compare the relative predictive power of adherence to the MD associated with the 25OHD levels, we performed a multiple linear regression analysis using a model that included PREDIMED score, sex, age, and BMI. Using this model, BMI entered at the first step (*p* < 0.001), followed by PREDIMED score, sex, and age. Results are reported in Table 6.

**Figure 3:**
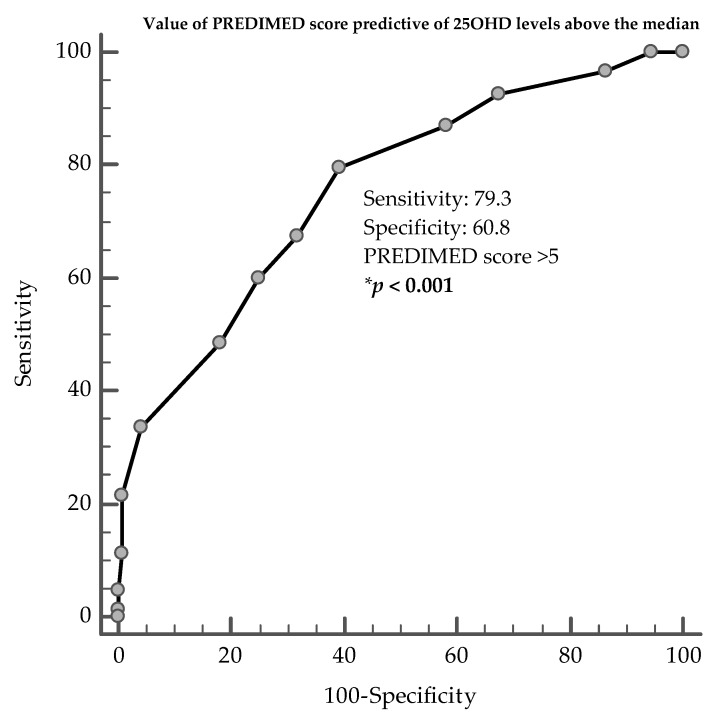
ROC for value of PREDIMED score predictive of 25OHD levels above the median, in males. * A *p*-value in bold type denotes a significant difference (*p* < 0.05).

**Figure 4:**
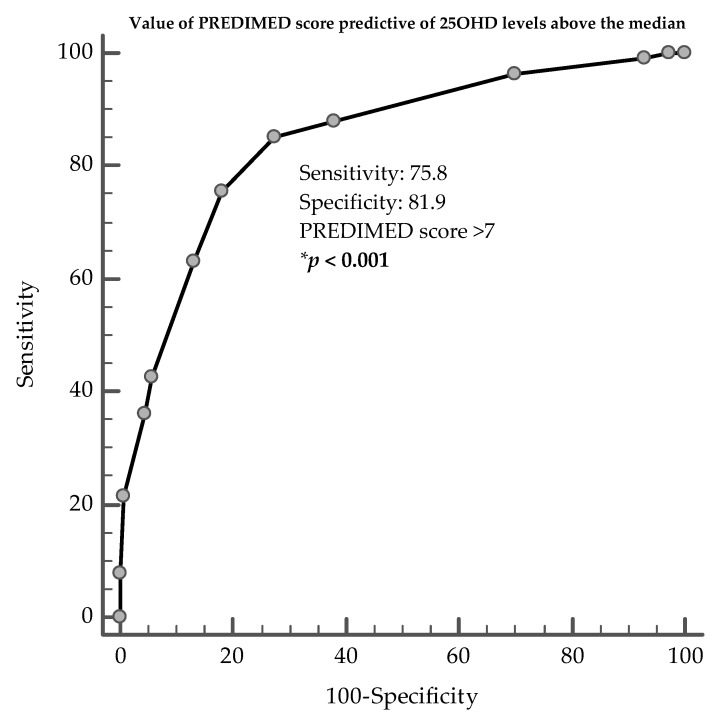
ROC for value of PREDIMED score predictive of 25OHD levels above the median, in females. * A *p*-value in bold type denotes a significant difference (*p* < 0.05).

ROC analysis was then performed to determine the cut-off value of PREDIMED score predictive of 25OHD levels. In particular, PREDIMED score >5 in males (*p* < 0.001, AUC 0.76, standard error 0.03, 95% CI 0.71 to 0.81; Figure 3), and >7 in females (*p* < 0.001, AUC 0.85, standard error 0.02, 95% CI 0.81 to 0.89; Figure 4) could serve as thresholds for 25OHD levels above the median (16.2 ng/mL and 16.9 ng/mL, in males and females, respectively).

## 4. Discussion

In this cross sectional, single center study, we reported a novel positive association between the adherence to the MD and the 25OHD levels, independent of BMI, in an adult population stratified according to gender across BMI categories. This stratification allowed us to confirm the presence of a gender difference either in the 25OHD levels or the adherence to the MD; although 25OHD levels were higher in males than females, 69.7% were deficient. In particular, compared to males, females exhibited higher adherence to the MD, despite lower 25OHD levels, with a lower percentage of 25OHD sufficiency as well. As expected, PREDIMED scores and 25OHD levels were the lowest in study participants with the highest grades of obesity (grade III). Nevertheless, the joint evaluation of PREDIMED score and 25OHD levels across different BMI categories in both genders had not previously been reported. As the highest values of PREDIMED score were reported in the subjects with the highest 25OHD levels and the lowest BMIs, these results suggested the key role of the adherence to the MD in both 25OHD levels and BMI. Based on the ROC curve analysis, the most sensitive and specific cut-off values for the PREDIMED score to predict the highest 25OHD levels were >5 and >7, in males and females, respectively. To date, to the best of our knowledge, this is the first study investigating gender differences in the relationship between adherence to the MD and 25OHD levels in a sample of an adult population across BMI categories.

Robust scientific evidence showed that 25OHD levels are lower in subjects with obesity than in normal-weight subjects [60,61,62]. The underlying mechanism of low 25OHD levels in subjects with obesity may be due to a number of factors, including low dietary vitamin D intake, little sun exposure, and low outdoor activity. In addition, accumulation of vitamin D in fat mass has been proposed as a potential mechanism [63]. Another hypothesis considers the result of volumetric dilution of vitamin D in the large adipose stores [64], as reported in women with obesity compared to normal-weight controls [65]. A recent meta-analysis of randomized controlled trials showed an overall inverse relationship between 25OHD levels and BMI in studies of non-diabetic subjects (r = −0.152, 95% = −0.187 to −0.116, *p* < 0.001), concluding that the vitamin D deficiency was associated with an increase in BMI [4].

Vitamin D receptors and vitamin D metabolizing enzymes are expressed in both adipocytes and inflammatory cells infiltrating adipose tissue depots. Beyond an anti-adipogenic effect, their presence also suggests an important role for vitamin D in regulating inflammatory pathways [66,67,68]. In this context, several piece of evidence showed that low vitamin D status was associated via low grade chronic inflammation, with consequent increased risk of metabolic and cardiovascular diseases [69,70]. Beyond its anti-inflammatory effects, the link between low 25OHD levels and risks of several diseases could be also partially explained by the antioxidant effects of vitamin D [71,72,73]. Of interest, in obese children, Codoñer-Franch P. et al. evidenced that a low vitamin D status was associated with high levels of oxidative stress biomarkers, including malondialdehyde, myeloperoxidase, 3-nitrotyrosine, interleukin (IL)-6, and soluble vascular cell adhesion molecule-(sVCAM)-1 [74]. According to these results, other studies confirmed the links among obesity, low vitamin D status, and increased oxidative stress [2,75].

The anti-inflammatory and antioxidant effects of the MD are equally well-known [76,77]. The MD pattern is the traditional diet of some of the countries of the Mediterranean basin that gathers the following characteristics: high consumption of whole grain, legumes, fruits, vegetables, nuts, and seeds; moderate intake of fish and shellfish, white meat, and eggs; and dairy products, red meat, processed meats, and foods rich in sugars in relatively small amounts. Moderate intake of wine, especially red wine with meals and extra virgin olive oil as the main source of fat, are also recommended [78,79,80]. From a nutrient point of view, MD is characterized by a relatively high fat consumption (40%–50% of total daily calories), by which between 15% and 25% of calories are represented by monounsaturated fatty acids, and ≤8% of calories are comprised of saturated fatty acids [81,82]. The favorable effects on health status of the MD are to be attributed also to high consumption of dietary fiber, food with low glycemic index, and glycemic load [83].

Among favorable effects of vitamin D, several studies reported that subjects with higher adherence to the MD also have higher dietary vitamin D intake [24,25,26], but only one study in adolescents reported that the adherence to the MD was positively associated with 25OHD levels [29]. However, in their study, the authors underlined a need to increase the knowledge on the nutritional adequacy of vitamins and the MD [23]. In addition, it is well known that gender is a well-known non-modifiable factor largely influencing food preferences and that women are more inclined to a Mediterranean dietary profile [31,32]. In our study, we confirmed that adult females had significantly lower 25OHD levels than adult males. However, it is evident from the gender difference in the association between the adherence to the MD and 25OHD levels in males, that despite a lower adherence to the MD compared to females, males have higher 25OHD levels. Although 25OHD levels were higher in males than females, 69.7% were deficient. As these results were independent of BMI, we hypothesized that the well-known sex difference in body fat and body fat distribution could likely account for this gender difference in the association between the two variables. Indeed, very recently we have demonstrated that females had significantly lower 25OHD concentrations than males among the different classes of BMI and that this sex difference was mainly explained by the higher fat mass percentage in women than men [37]. However, the different intakes of the foods of the MD between females and males could provide a further explanation. In our study the extra virgin olive oil consumption was different in males than in females, and it is known that dietary fats may change vitamin D absorption [84]. In particular, it has been reported that an increased consumption of monounsaturated fatty acids-rich oils, such as olive oil, may improve the bioavailability of vitamin D [85].

Of interest, although there is some disagreement [19], both a meta-analysis by Esposito et al. including 3436 participants [20], and a more recent systematic review of five randomized clinical trials indicated that a MD, in combination with physical activity, MD is effective at inducing weight loss, especially when it was energy restricted, and lasted longer than six months [16]. In particular, the high content of polyphenols in the MD could exert favorable its effects on weight loss through different mechanisms, including activation of β-oxidation, prebiotic effects for gut microbiota, induction of satiety, and modulation of both brown and white adipose tissue [19].

Taking into account the independent roles of adherence to the MD and vitamin D in obesity, the translational potential of this study suggests that the high adherence to the MD, likely due its anti-inflammatory and antioxidant properties, might contribute to offerring a unique advantage in the management of subjects with low vitamin D status and obesity. In addition, gender-specific cut-off values for the PREDIMED score might help to predict the risk of the lowest levels of 25OHD levels in adults.

We are aware that there are some limitations in the current study.

First, the cross-sectional design of this study did not allow for any statements on a causal relationship between adherence to the MD and 25OHD levels. Moreover, the suggested cut-offs values of PREDIMED score should be viewed with caution until results of studies in larger patient populations have become available to perform an appropriate cross-validation.

Second, the possible underlying inflammatory and antioxidant status linking PREDIMED score, 25OHD levels, and obesity, should be better investigated by measuring both serum inflammatory and antioxidant biomarkers, such as IL-6, c-reactive protein levels, superoxide dismutase, glutathione peroxidase, catalase, and malondialdehyde; thus, our hypothesis of a synergistic effect of adherence to the MD and 25OHD levels on obesity remains largely speculative.

Third, we did not include the measurements of the body composition in this study.

Fourth, the adherence to the MD was assessed with the 14-items questionnaire of the PREDIMED study [52]. This questionnaire is less expensive, less time-demanding, and requires less collaboration from participants than the usual full-length food frequency questionnaire or other more comprehensive methods [52], with which it was previously validated [86]. In addition, the PREDIMED questionnaire allows one to provide feedback to the participant immediately after the interview is completed. However, the design of this questionnaire of 14-items does not allow for an evaluation of total energy and dietary intake, and particularly, dietary vitamin D intake. This evaluation must be done with the seven-day food records, as it is the gold standard and allows for accurate measurements of the real dietary and macronutrient intakes compared to those that were obtained by retrospective food frequency questionnaires [87]. Therefore, we could only speculate that the observed lower adherence to the MD in males compared to females, despite their higher 25OHD levels, could be the epiphenomenon of the well-known sex difference in body fat and body fat distribution.

Fifth, sunlight exposure time have not been sufficiently evaluated. Sun is the major causal factor for melanoma, and melanoma incidence rates in Italian men (40.4 *per* 100,000) are much higher than in Italian women (11.0 *per* 100,000) [88]. This would therefore indicate that sun exposure in Italian males is higher than in Italian females. Sun exposure is also the main predictor of circulating 25OHD levels [59,89,90]; only a small proportion of circulating 25OHD levels are attributable to diet (excluding supplementation) [7]. In this study, we did not evaluate the sun-exposure habits. Thus, the role of sun exposure in influencing the difference in 25OHD levels between males and females can not be excluded.

Nevertheless, a major strength of this study includes the very large sample of subjects stratified by gender—making possible the comparisons between the study variables across different categories of BMI, PREDIMED, and 25OHD independently of gender. In addition, our study was based on a single clinical center, with a possibility of selection bias in the results.

However, the single-center study allowed us to increase the homogeneity of the sample as we included subjects evaluated in the same season and living in the same geographical area around Naples metropolitan area, Italy (latitude 40° 49′ N; elevation 17 m), so with the same effect of latitude on vitamin D levels, and likely, with similar nutrient availability. Finally, the PREDIMED questionnaire was face-to-face administered and not self-reported in order to minimize any bias related to the filling of the questionnaire, and to avoid the inter-operator variability, only one expert Nutritionist evaluated the anthropometric measures and administered the PREDIMED questionnaire.

## 5. Conclusions

In summary, the results of our study reported a novel positive association between adherence to the MD and 25OHD levels in an adult population both gender across BMI categories, with a gender-specific difference in this association. As possible translational applications, these findings: (i) suggest the advantage of possible synergistic anti-inflammatory and antioxidant effects of MD and vitamin D; (ii) provide gender-specific cut-off values for the PREDIMED score to predict the risk of the lowest 25OHD levels in adults; (iii) recommend the dietary assessment, and in particular, the evaluation of adherence to the MD, as good clinical practice in the management of patients with low vitamin D status and obesity. Further clinical trials on large series populations will be of paramount importance to elucidate the potential translational applications of the results of this study and the beneficial effects of MD as a medical prescription for low vitamin D status and obesity.

## Figures and Tables

**Table 1:** Age, anthropometric characteristics, adherence to the MD, and 25OHD levels in the whole study population and stratified by gender.

Parameters.	All Participants Mean ± SD or Number (%) n = 617	Males Mean ± SD or Number (%) n = 296, 48.0%	Females Mean ± SD or Number (%) n = 321, 52.0%	* p-Value
Age (years)	36.5 ± 11.0	36.8 ± 11.2	36.1 ± 10.9	0.48
BMI (kg/m^2^)	34.3 ± 8.0	34.3 ± 8.2	34.4 ± 7.9	0.95
Normal-weight	96, 15.6%	47, 15.9%	49, 15.3%	χ^2^ = 0.01, *p* = 0.92
Overweight	109, 17.7%	52, 17.6%	57, 17.8%	χ^2^ = 0.01, *p* = 0.96
Grade I obesity	121, 19.6%	57, 19.3%	64, 19.9%	χ^2^ = 0.01, *p* = 0.91 χ^2^ = 2.92, *p* = 0.08
Grade II obesity	127, 20.6%	70, 23.6%	57, 17.8%	
Grade III obesity	164, 26.6%	70, 23.6%	94, 29.3%	χ^2^ = 2.22, *p* = 0.14
PREDIMED (score)	7.0 ± 3.0	6.7 ± 3.1	7.4 ± 2.8	**0.001**
Low adherence to the MD	238, 38.6%	119, 40.2%	119, 37.1%	χ^2^ = 8.94, ***p* = 0.003**
Average adherence to the MD	245, 39.7%	121, 40.9%	124, 38.6%	χ^2^ = 0.62, *p* = 0.06
High adherence to the MD	134, 21.7%	56, 18.9%	78, 24.3%	χ^2^ = 2.30, *p* = 0.12
25OHD levels	17.5 ± 7.6	18.3 ± 7.3	16.8 ± 7.8	**0.01**
Deficit	413, 66.9%	206, 69.6%	207, 64.5%	χ^2^ = 1.59, *p* = 0.21
Insufficiency	162, 26.3%	59, 19.9%	103, 32.1%	χ^2^ = 11.13, ***p* < 0.001**
Sufficiency	42, 6.8%	31, 10.5%	11, 3.4%	χ^2^ = 10.96, ***p* < 0.001**

* A *p* value in bold type denotes a significant difference (*p* < 0.05) between males and females.

**Table 2:** The PREDIMED score and 25OHD levels in the population study across BMI categories.

BMI Categories.	PREDIMED (Score)	25OHD Levels (ng/mL)
Normal-weight	9.8 ± 3.1	24.8 ± 6.5
Overweight	8.6 ± 2.7	22.6 ± 6.5
Grade I obesity	7.4 ± 2.5	18.5 ± 6.3
Grade II obesity	6.3 ± 2.2	14.3 ± 5.1
Grade III obesity	4.8 ± 1.9	11.6 ± 4.8
* *p*-value	**<0.001**	**<0.001**

* A *p* value denotes a significant difference (*p* < 0.05).

**Table 3:** Response frequencies of dietary components included in the PREvención con DIetaMEDiterránea (PREDIMED) questionnaire in the study population according to gender.

Questions of PREDIMED Questionnaire	Males		Females			
	n	%	n	%	χ	* p-Value
**Use of extra virgin olive oil as main culinary lipid**	231	78.0	224	69.8	5.00	**0.03**
**Extra virgin olive oil >4 tablespoons**	149	50.3	138	43.0	3.05	0.08
**Vegetables ≥2 servings/day**	139	47.0	180	56.1	4.07	**0.04**
**Fruits ≥3 servings/day**	162	54.7	205	63.9	4.96	**0.03**
**Red/processed meats <1/day**	96	32.4	191	59.5	44.27	**<0.001**
**Butter, cream, margarine <1/day**	171	57.8	177	55.1	0.33	0.56
**Soda drinks <1/day**	131	44.3	182	56.7	9.05	**0.003**
**Wine glasses ≥7/week**	175	59.1	107	66.7	40.23	**<0.001**
**Legumes ≥3/week**	159	53.7	211	65.7	8.77	**0.003**
**Fish/seafood ≥3/week**	148	50.0	171	53.3	0.54	0.46
**Commercial sweets and confectionery ≤2/week**	114	38.5	161	50.2	7.98	**0.005**
**Tree nuts ≥3/week**	57	19.3	64	19.9	0.012	0.91
**Poultry more than red meats**	128	43.2	189	58.9	14.45	**<0.001**
**Use of sofrito sauce ≥2/week**	111	37.5	178	54.8	18.53	**<0.001**

* A *p* value in bold type denotes a significant difference (*p* < 0.05) between males and females.

**Table 4:** Descriptive data of the 25OHD categories and PREDIMED scores according to PREDIMED categories and stratified by gender.

Parameters	Males Number (%) or Mean ± SD n = 296, 48.0%	Females Number (%) or Mean ± SD n = 321, 52.0%	* *p*-Value
Low adherence to the MD category			χ^2^ = 1.01, *p* = 0.31
25OH deficiency	102, 85.7%	108, 90.8%	
25OH insufficiency/normal levels	17, 14.3%	11, 9.2%	
PREDIMED score	3.5 ± 1.3	4.4 ± 0.8	**<0.001**
Average adherence to the MD category			χ^2^ = 0.69, *p* = 0.41
25OH deficiency	86, 71.1%	81, 65.3%	
25OH insufficiency/normal levels	35, 29.8%	43, 34.7%	
PREDIMED score	7.7 ± 1.2	7.9 ± 1.1	0.48
High adherence to the MD category			χ^2^ = 0.94, *p* = 0.33
25OH deficiency	18, 32.1%	18, 23.1%	
25OH insufficiency/normal levels	38, 67.9%	60, 76.9%	
PREDIMED score	11.1 ± 1.1	11.5 ± 0.9	**0.03**

* A *p* value in bold type denotes a significant difference (*p* < 0.05) between males and females.

**Table 5:** Bivariate proportional odds ratio (OR) model to assess the association between 25OHD levels and PREDIMED categories.

Categories.	OR	Wald Test	Standard Error	*p*-Heterogeneity and *p*-Trend	95% IC	R^2^
	**Males, n = 296, 48.0%**					
Low adherence to the MD category vs. Average adherence to the MD category	1.13	23.24	0.026	<0.001	1.08–1.19	0.120
Low adherence to the MD category vs. high adherence to the MD category	1.21	37.95	0.031	<0.001	1.14–1.29	0.284
Average adherence to the MD category vs. high adherence to the MD category	1.10	15.37	0.024	<0.001	1.04–1.49	0.089
	**Females, n = 321, 52.0%**					
Low adherence to the MD category vs. Average adherence to the MD category	1.16	41.62	0.024	<0.001	1.11–1.22	0.203
Low adherence to the MD category vs. high adherence to the MD category	1.31	60.50	0.035	<0.001	1.22–1.40	0.462
Average adherence to the MD category vs. high adherence to the MD category	1.17	29.58	0.028	<0.001	1.10–1.23	0.174

**Table 6:** Multiple regression analysis models (stepwise method) with the 25OHD level as the dependent variable to estimate the predictive value of PREDIMED score, sex, age, and BMI.

Parameters	Multiple Regression Analysis			
	R^2^	β	t	* p Value
**BMI (kg/m^2^)**	0.44	–0.67	–22.1	**<0.001**
**PREDIMED score**	0.47	0.21	5.6	**<0.001**
**Sex (Males/Females)**	0.49	–0.13	–4.4	**<0.001**
**Age (years)**	0.49	–0.09	–2.9	**0.003**

* A *p* value in bold type denotes a significant difference (*p* < 0.05).

## Abbreviations

**25OHD**	25-hydroxyvitamin D
**MD**	Mediterranean diet
**BMI**	body mass index
**PREDIMED**	Prevención con dieta Mediterránea
**CVs**	coefficients of variation
**OR**	Proportional odds ratio
**IC**	interval confidence
**ROC**	receiver operator characteristic
**AUC**	area under the curve
**IL**	interleukin
**sVCAM**	soluble vascular cell adhesion molecule
